# Identification of TBK1 complexes required for the phosphorylation of IRF3 and the production of interferon β

**DOI:** 10.1042/BCJ20160992

**Published:** 2017-03-15

**Authors:** Siddharth Bakshi, Jordan Taylor, Sam Strickson, Thomas McCartney, Philip Cohen

**Affiliations:** MRC Protein Phosphorylation and Ubiquitylation Unit, School of Life Sciences, University of Dundee, Dundee DD1 5EH, U.K.

**Keywords:** interferon, IRF3, LPS, TBK1, TLR3, ubiquitin

## Abstract

The double-stranded RNA mimetic poly(I:C) and lipopolysaccharide (LPS) activate Toll-like receptors 3 (TLR3) and TLR4, respectively, triggering the activation of TANK (TRAF family member-associated NF-κB activator)-binding kinase 1 (TBK1) complexes, the phosphorylation of interferon regulatory factor 3 (IRF3) and transcription of the interferon β (IFNβ) gene. Here, we demonstrate that the TANK–TBK1 and optineurin (OPTN)–TBK1 complexes control this pathway. The poly(I:C)- or LPS-stimulated phosphorylation of IRF3 at Ser396 and production of IFNβ were greatly reduced in bone marrow-derived macrophages (BMDMs) from TANK knockout (KO) mice crossed to knockin mice expressing the ubiquitin-binding-defective OPTN[D477N] mutant. In contrast, IRF3 phosphorylation and IFNβ production were not reduced significantly in BMDM from OPTN[D477N] knockin mice and only reduced partially in TANK KO BMDM. The TLR3/TLR4-dependent phosphorylation of IRF3 and IFNβ gene transcription were not decreased in macrophages from OPTN[D477N] crossed to mice deficient in IκB kinase ε, a TANK-binding kinase related to TBK1. In contrast with the OPTN–TBK1 complex, TBK1 associated with OPTN[D477N] did not undergo phosphorylation at Ser172 in response to poly(I:C) or LPS, indicating that the interaction of ubiquitin chains with OPTN is required to activate OPTN–TBK1 in BMDM. The phosphorylation of IRF3 and IFNβ production induced by Sendai virus infection were unimpaired in BMDM from TANK KO × OPTN[D477N] mice, suggesting that other/additional TBK1 complexes control the RIG-I-like receptor-dependent production of IFNβ. Finally, we present evidence that, in human HACAT cells, the poly(I:C)-dependent phosphorylation of TBK1 at Ser172 involves a novel TBK1-activating kinase(s).

## Introduction

Type 1 interferons (IFNs) have critical roles in host defence against viral and bacterial pathogens. The engagement of Toll-like receptor 3 (TLR3) by viral double-stranded (ds) RNA, or TLR4 by bacterial lipopolysaccharide (LPS), triggers the production of IFNβ by a signalling network that requires, first, the recruitment of the adaptor protein TRIF (TIR-domain-containing adapter-inducing IFN-β, also known as TICAM1) [1], second, the activation of the IκB kinase-related enzymes TBK1 [TRAF family member-associated NF-κB activator (TANK)-binding kinase 1] [2] and IκB kinase ε (IKKε) [3–5] and, third, the phosphorylation of interferon regulatory factor 3 (IRF3) [6]. The phosphorylation of IRF3 at C-terminal serine residues, such as Ser396 [7,8], induces its dimerization and translocation to the nucleus where it binds to the promoter of the *IFNβ* gene and stimulates transcription [9–12]. Other TBK1-regulated proteins, such as the transcription factor Deformed Epidermal Auto-regulatory Factor 1 [13] and the RNA helicase DDX3X [14,15], may also stimulate TLR3/TLR4-dependent IFNβ gene transcription.

The critical importance of this pathway *in vivo* has been revealed by the identification of mutations in the genes encoding TLR3, TRIF, TBK1 or IRF3 that impair IFNβ production and underlie Herpes simplex virus encephalitis, a devastating disease of the central nervous system in young children [16,17]. On the other hand, the overproduction of IFNβ by the TLR4/TRIF signalling network causes endotoxaemia and endotoxic shock, since IFNβ knockout (KO) mice or mice lacking expression of the type1 IFN receptor are resistant to LPS-induced sepsis [18–20].

The RNA helicases RIG-I and MDA-5 also recognize RNA molecules formed during the replication of single-stranded RNA viruses [21–23] and trigger the activation of TBK1, the phosphorylation of IRF3 and IFNβ gene transcription. However, these RIG-I-like receptors (RLRs) do not signal via TRIF, but by the adaptor termed mitochondrial antiviral signalling protein (MAVS; also known as interferonβ promoter stimulator-1; virus-induced-signaling adapter and CARD adapter-inducing interferonβ) [24–27].

More recently, TBK1 was reported to have a dual role in these pathways. First, it phosphorylates TRIF and MAVS [28], which permit the interaction of the transcription factor IRF3 with these proteins; second, it phosphorylates IRF3 at amino acid residues that include Ser396 [29]. One attractive feature of this mechanism is that it can explain why the activation of TLR3 and TLR4, but not the activation of TLRs that signal via the adaptor MyD88 (myeloid differentiation primary response gene 88), triggers IRF3 phosphorylation [28], even though TBK1 is activated robustly when any TLR is activated [30].

TBK1 does not exist as a single entity in cells but as a variety of heterodimers in which it forms complexes with TANK [2], NF-κB-activating kinase-associated protein 1 (NAP1) [31], SINTBAD (Similar to NAP1 TBK1 Binding Adaptor) [32] and the ubiquitin-binding protein optineurin (OPTN) [33]. IKKε also interacts with TANK [34], NAP1 [31] and SINTBAD [32], but not with optineurin [33,35]. NAP1 was reported to interact with TRIF, whereas TANK did not [36], and RNA interference studies suggested that NAP1 was required for both TLR3-dependent and RIG-I/MDA-5-dependent phosphorylation of IRF3 and IFNβ production [36,37]. The shRNA knockdown of NAP1, SINTBAD or TANK also led to decreases in Sendai virus-induced IFNβ gene transcription in overexpression studies performed in human 293 cells. However, studies with TANK KO mice failed to find any involvement of TANK in viral responses, but instead revealed that it was a negative regulator of TLR signalling. As a consequence, TANK KO mice overexpressed proinflammatory cytokines and developed autoimmune nephritis, which could be prevented by crossing to MyD88 KO mice [38]. Subsequently, the TANK–TBK1 and TANK–IKKε complexes were found to phosphorylate the catalytic and regulatory subunits of the canonical IKK complex on sites that inhibit their catalytic activity, explaining how TANK restricts MyD88 signalling [30,34]. TANK plays a key role in facilitating this process via its interaction with the NEMO (NF-κB essential modulator) component of the IKK complex [39,40].

In the present paper, we have reinvestigated the roles that TANK and optineurin have in TLR3- and TLR4-dependent IFNβ production after crossing TANK KO mice with knockin mice in which optineurin is replaced by a ubiquitin-binding-defective mutant. These studies have demonstrated that the TANK–TBK1 and OPTN–TBK1 complexes both participate in these signalling networks.

## Materials and methods

### Materials

MRT67307, a potent inhibitor of TBK1 and IKKε [30], was dissolved in dimethyl sulphoxide and stored at −20°C as a 10 mM solution. LPS (*Escherichia coli* strain O5:B55) was from Alexis Biochemicals (ALX-581-013-L002), and poly(I:C) from InvivoGen (tlrl-pic). macrophage colony-stimulating factor (M-CSF) was purchased from R&D Systems (216-MC-025) and FuGene® HD transfection reagent from Promega (E2311).

### Antibodies

Antibodies for immunoprecipitation were raised in sheep against amino acid residues 520–531 of human OPTN (sheep number S685D, fourth and fifth bleeds) and the full-length human TANK protein (sheep number S278C, third bleed), and were generated by the antibody production team of the Medical Research Council Protein Phosphorylation and Ubiquitylation Unit, University of Dundee (co-ordinated by Dr James Hastie). They can be ordered from the reagents section of the MRC-PPU website (https://mrcppureagents.dundee.ac.uk/). The following antibodies for immunoblotting were purchased from Cell Signaling Technology: TANK (Cat #2141), TBK1 (Cat #3504), TRIF (Cat #4596), IKKε (Cat #3416), GAPDH (Cat #2118), c-Jun N-terminal kinase (JNK)1/2 (Cat #9258), p38 (Cat #9212), TBK1 phosphorylated at Ser172 (Cat #5483), IKKε phosphorylated at Ser172 (Cat #8766), TAK1 (TGFβ-activated kinase 1; Cat #4505), IKKα phosphorylated at Ser176 and Ser180 and IKKβ phosphorylated at Ser177 and Ser181 (Cat #2677), IRF3 phosphorylated at Ser396 (Cat #4947), JNK1 and JNK2 dually phosphorylated at their Thr-Pro-Tyr motifs (Cat #4668) and p38α mitogen-activated protein (MAP) kinase dually phosphorylated at its Thr-Gly-Tyr motif (Cat #9211). An anti-OPTN antibody was obtained from Abcam (Cat #ab23667), an antibody that recognizes all forms of IRF3 was from Proteintech (Cat #11312-1-AP) and an antibody that recognizes all forms of IKKβ was from Merck-Millipore (Cat #05-535). Rabbit- and sheep-specific secondary antibodies conjugated to horseradish peroxidase were from Thermo Scientific.

### Mice, cell culture, cell stimulation and cell lysis

Heterozygous TANK KO mice (a gift from Professor Shizuo Akira, Laboratory of Host Defense, World Premier International Immunology Frontier Research Center, Osaka University, Japan) [38] and IKKε KO mice (a gift from Dr Alastair Reith, GlaxoSmithKline, Stevenage, U.K.) were crossed to OPTN[D477N] mice [35] to generate TANK KO × OPTN[D477N] and IKKε KO × OPTN[D477N] mice, respectively. BMDMs were obtained by differentiating bone marrow obtained from the femur and tibia with M-CSF or L929 preconditioned medium as the source of M-CSF [41]. Adherent BMDMs were re-plated into 12-well tissue culture plates (5 × 10^5^ cells/well) or 10 cm tissue culture grade plates (5 × 10^6^ cells/plate) using fresh culture medium. After re-plating, the BMDMs were stimulated with the TLR ligands indicated in the figure legends. The human keratinocyte HACAT cell line was cultured in Dulbecco's Modified Eagle's medium supplemented with 10% foetal bovine serum, 2 mM l-glutamine and antibiotics (100 Units/ml penicillin and 0.1 mg/ml streptomycin). Where indicated, cells were incubated for 1 h with MRT67307 dissolved in dimethyl sulphoxide or an equivalent volume of dimethyl sulphoxide for control incubations.

The cells were rinsed in ice-cold PBS and extracted in ice-cold lysis buffer [50 mM Tris–HCl (pH 7.5), 1 mM EGTA, 1 mM EDTA, 1% (v/v) Triton X-100, 1 mM sodium orthovanadate, 50 mM sodium fluoride, 5 mM sodium pyrophosphate, 0.27 M sucrose, 10 mM sodium 2-glycerophosphate, 1 mM phenylmethylsulphonyl fluoride, 1 mM benzamidine and 1 mM dithiothreitol]. Cell lysates were clarified by centrifugation at 14 000×***g*** for 30 min at 4°C, and the supernatants (cell extracts) were collected and their protein concentrations were determined by the Bradford procedure.

### Generation of TRIF- or TAK1-deficient HACAT cells

HACAT cells (2 × 10^6^ cells) were plated onto a 10 cm diameter tissue culture grade plate and transfected the next day with 1 µg each of guide (g) RNA plasmid targeting the gene of interest. FuGene® HD transfection reagent (5 µl per 1 µg of plasmid DNA) was used for transfection. At 24 and 48 h post-transfection, fresh media containing 2 µg/ml puromycin was added to the cells, while 72 h after transfection the puromycin-containing medium was replaced by medium lacking puromycin. After a further 48 h, cells were single-cell plated onto 96-well plates and left for 2–3 weeks until colonies began to form. The colonies were then analyzed for the expression of TRIF and TAK1 by immunoblotting.

### Immunoblotting and immunoprecipitation

Immunoblotting was performed using the ECL detection system (GE Healthcare). To immunoprecipitate TANK and OPTN, 1.0 mg of cell extract protein was incubated for 2 or 4 h with 3.0 µg of anti-TANK or 6.0 µg of anti-OPTN. Protein-G-Sepharose beads (20 µl) were added and, after end-over-end rotation for 1 h at 4°C, the beads were collected by centrifugation, washed three times with cell lysis buffer, denatured in SDS, subjected to SDS–PAGE, transferred to polyvinylidene fluoride membranes and immunoblotted.

### Quantitative RT-PCR and ELISA

Total RNA was extracted from macrophages using the MicroElute Total RNA kit (Omega bio-tek). RNA was reverse-transcribed using the iScript cDNA synthesis kit (Bio-Rad) following the manufacturer's instructions. Polymerase chain reaction (PCR) mixes were assembled using the SsoFast™ EvaGreen® Supermix (Bio-Rad). Reactions were performed with the SYBR Green (plus melting curve analysis) programme on the C1000 thermal cycler quantitative PCR system (Bio-Rad). All reactions were performed in duplicate. The concentration of IFNβ released into the cell culture medium was determined by using the LegendMax mouse IFNβ ELISA kit (Biolegend). The primer sequences used in the present paper are given in Supplementary Table S1.

### Quantitation of immunoblots of phosphorylated IRF3

This was performed using the Image J software and normalized to immunoblots obtained with antibodies that recognize all forms of IRF3.

### Statistical analyses

Statistical analyses were performed using the GraphPad Prism software. Quantitative data were presented as the arithmetic mean ± SEM. Statistical significance of differences between wild-type and the other genotypes was assessed using two-way ANOVA with Bonferroni post-test to compare each genotype with the control wild-type sample, unless stated otherwise. Differences in means were considered significant if *P* < 0.05; *P < 0.05, **P < 0.01, ***P < 0.001. n.s. means not significant.

## Results

### TANK–TBK1 and OPTN–TBK1 control TLR3/4-dependent phosphorylation of IRF3 and IFNβ gene transcription in BMDMs

**Figure 1.**
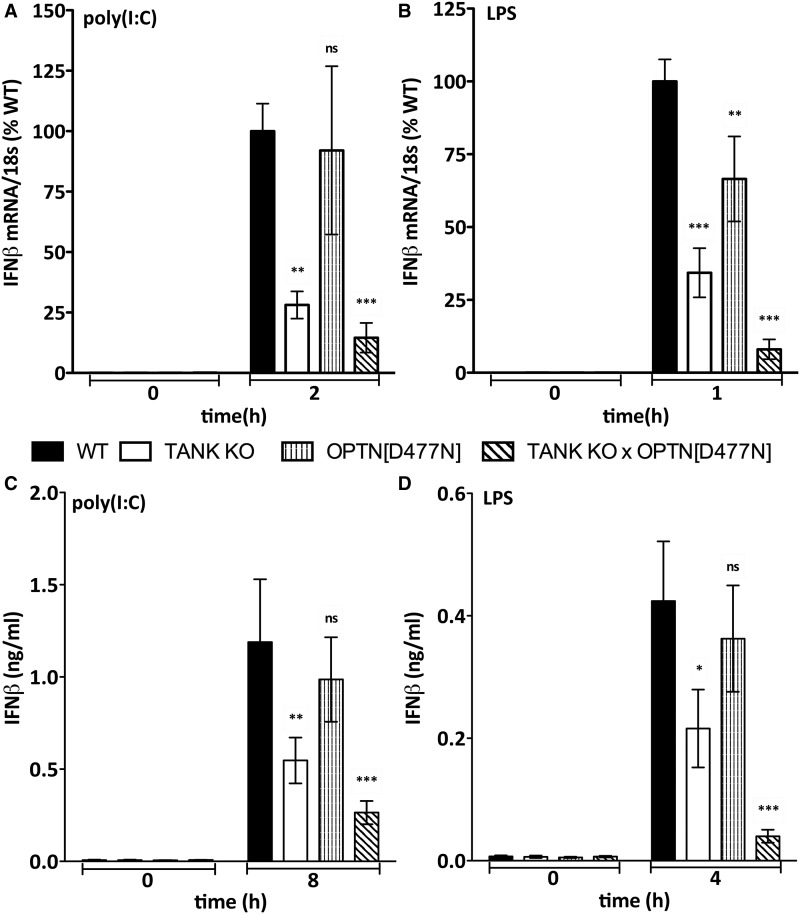
IFNβ production is suppressed in BMDM from TANK KO × OPTN[D477N] mice. BMDM from wild-type (WT) mice (closed bars), TANK KO mice (open bars), OPTN[D477N] mice (striped bars) and TANK KO × OPTN[D477N] mice (hatched bars) were stimulated with 10 µg/ml poly(I:C) (**A** and **C**) or 100 ng/ml LPS (**B** and **D**) for the times indicated. (**A** and **B**) The IFNβ mRNA produced was measured relative to 18S ribosomal mRNA and normalized to the level of wild-type IFNβ mRNA (100%) measured after 2 h stimulation with poly(I:C) (**A**) or 1 h stimulation with LPS (**B**). (**C** and **D**) The amount of IFNβ in the cell culture medium was measured by ELISA after 8 h stimulation with poly(I:C) (**C**) or 4 h stimulation with LPS (**D**). (**A**–**D**) The results are presented as arithmetic mean (±SEM for four independent experiments carried out on BMDM from nine different mice).

**Figure 2.**
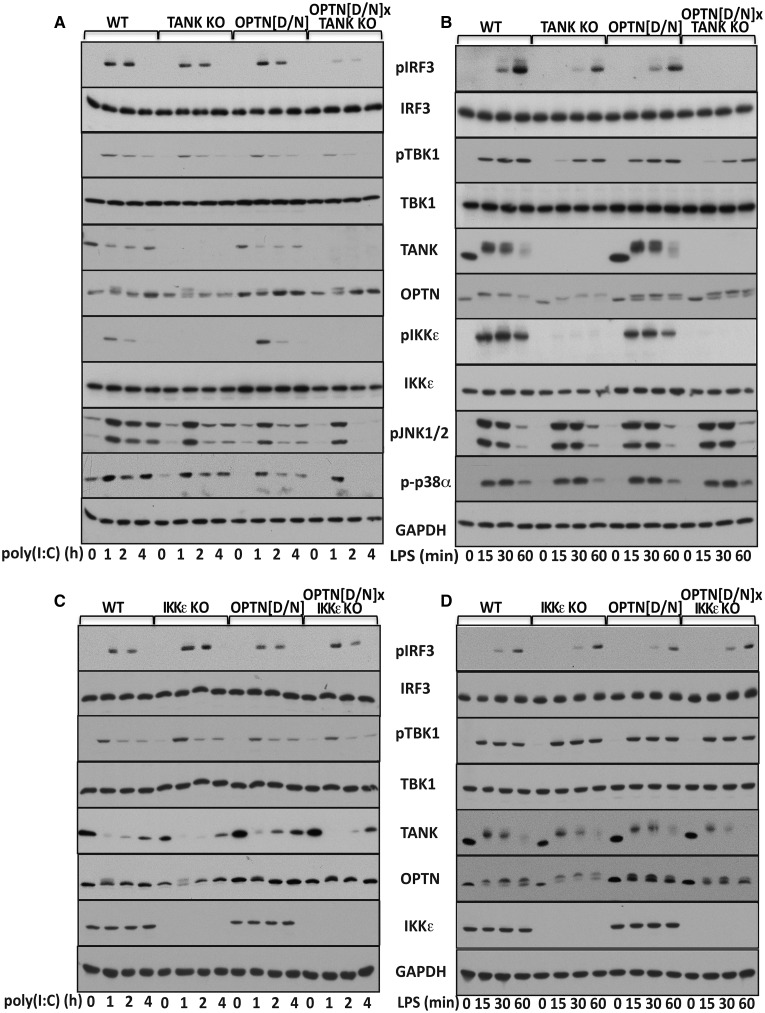
Poly(I:C)- or LPS-induced phosphorylation of TBK1, IRF3 and MAP kinases in BMDM from different mouse lines. (**A**–**D**) BMDM from WT mice and TANK KO mice (**A** and **B**), IKKε KO mice (**C** and **D**), OPTN[D477N] mice and either OPTN[D/N] × TANK KO mice (**A** and **B**) or OPTN[D/N] × IKKε KO mice (**C** and **D**) were stimulated with 10 µg/ml poly(I:C) or 100 ng/ml LPS for the times indicated. Aliquots of the cell extracts (20 µg protein) were subjected to SDS–PAGE and immunoblotted with antibodies that recognize TBK1 or IKKε phosphorylated at Ser172 (pTBK1 and pIKKε, respectively), IRF3 phosphorylated at Ser396 (pIRF3), JNK1 and JNK2 phosphorylated at their Thr-Pro-Tyr motifs (p-JNK1/2) and p38α phosphorylated at its Thr-Gly-Tyr motif (p-p38α), and with antibodies that recognize all forms of TBK1, IKKε, IRF3, TANK and OPTN. Antibodies to GAPDH were used as a loading control.

OPTN is the protein with the greatest amino acid sequence similarity to NEMO, the regulatory component of the canonical IKK complex. The proteins have similar ubiquitin-binding domains and, like NEMO, OPTN binds to Met1-linked ubiquitin (M1-Ub) and Lys63-linked ubiquitin (K63-Ub) chains, but not to Lys48-linked ubiquitin chains [35]. To investigate which TBK1 complexes are required for IRF3 phosphorylation and IFNβ production, we crossed knockin mice expressing the ubiquitin-binding-defective OPTN[D477N] mutant [35] to TANK KO mice [38], generating TANK KO × OPTN[D477N] mice. Stimulation of BMDMs with the dsRNA-mimetic poly(I:C) to activate TLR3, or LPS to activate TLR4, induced the production of IFNβ mRNA (Figure 1A,B) and secretion of IFNβ (Figure 1C,D), which were partially reduced in BMDM from the TANK KO, not reduced significantly in BMDM from OPTN[D477N] mice, but greatly reduced in BMDM from the TANK KO × OPTN[D477N] mice. Consistent with these findings, the TLR3-dependent phosphorylation of IRF3 at Ser396 was also reduced considerably in BMDM from the TANK KO × OPTN[D477N] mice (top two panels of Figure 2A and Supplementary Figure S1A) and the TLR4-dependent phosphorylation of IRF3 was undetectable (top two panels of Figure 2B and Supplementary Figure S1B). Consistent with these observations, the LPS-dependent secretion of IFNβ was reduced more strikingly than the poly(I:C)-stimulated secretion of IFNβ in BMDM from TANK KO × OPTN[D477N] mice. However, the TLR3- or TLR4-dependent phosphorylation of TBK1 at Ser172 was only reduced modestly in BMDM from TANK KO × OPTN[D477N] mice (Figure 2A,B, panels 3 and 4), indicating that poly(I:C) and LPS activate other TBK1 complexes distinct from TANK–TBK1 and OPTN–TBK1.

TLR3 signals specifically via the adaptor protein TRIF, whereas TLR4 signals via MyD88 and TRIF. The decreased phosphorylation of TBK1 at Ser172 observed 15 min after stimulation with LPS in BMDM from TANK KO and TANK KO × OPTN[D477N] mice is explained by the TANK–TBK1 complex making a major contribution to the MyD88-dependent activation of TBK1 [34], which occurs more rapidly than the TRIF-dependent activation of TBK1. Since only the slower TRIF-dependent activation of TBK1 induces IRF3 phosphorylation at Ser396, this explains why the LPS-dependent phosphorylation of TBK1 precedes the phosphorylation of IRF3.

The mRNAs encoding TLR3, TLR4 and TRIF were not decreased in BMDM from the TANK KO, OPTN[D477N] or TANK KO × OPTN[D477N] mice (Supplementary Figure S2A–C), indicating that the decreased phosphorylation of IRF3 and reduced production of IFNβ mRNA in BMDMs from TANK KO × OPTN[D477N] mice were not explained by decreased expression of these receptor and adaptor molecules that are situated ‘upstream’ of IRF3 in this signalling pathway. The expression of UNC93B, which interacts with TLR3 and localizes it to endosomal membranes [42], was also unimpaired in the TANK KO × OPTN[D477N] knockin mice (Supplementary Figure S2D). The expression of IRF3 in BMDM from TANK KO × OPTN[D477N] mice was similar to that from wild-type mice, while the expression of TANK was similar in BMDM from OPTN[D477N] mice and the expression of OPTN was similar in TANK KO mice (Figure 2A,B).

IKKε, the protein kinase most closely related to TBK1, also forms a complex with TANK [34] and has been reported to contribute to TLR3 and TLR4-dependent IFNβ production in some cells (see Introduction). However, the poly(I:C)- or LPS-stimulated phosphorylation of IRF3 at Ser396 (Figure 2C,D, top two panels), IFNβ mRNA production and IFNβ secretion (Figue 3A–D) were not impaired in macrophages from the OPTN[D477N] × IKKε KO mice. Indeed, IFNβ production was modestly elevated in the IKKε-deficient macrophages compared with wild-type macrophages. Taken together, these experiments indicate that the OPTN–TBK1 and TANK–TBK1 complexes are required for the TLR3- and TLR4-dependent phosphorylation of IRF3 at Ser396 and the production of IFNβ in BMDM.

**Figure 3.**
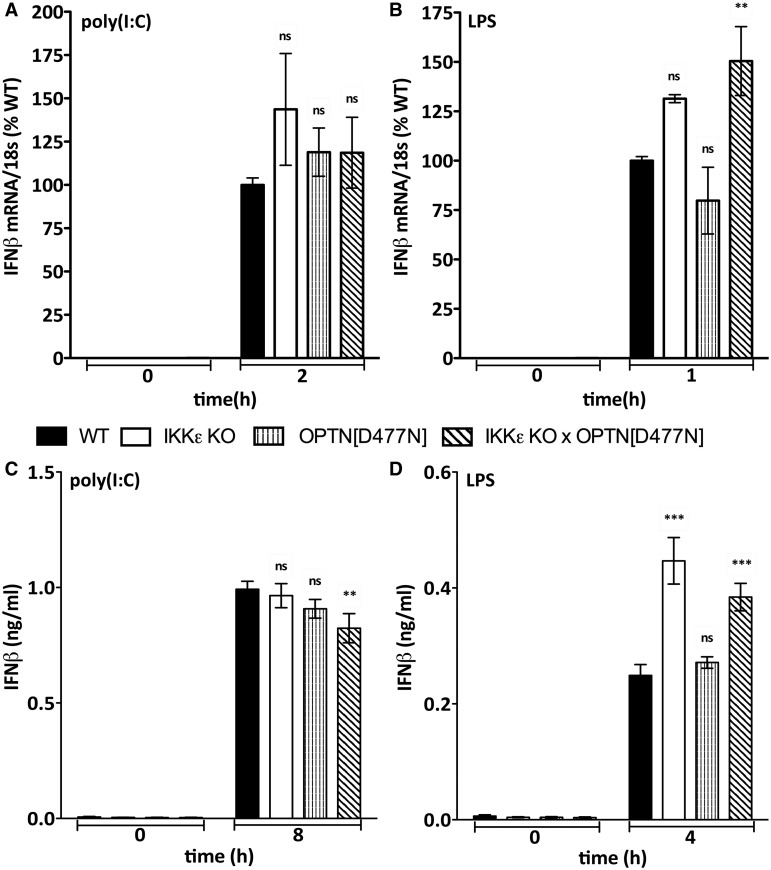
IFNβ production is not suppressed in BMDM from IKKε × OPTN[D477N] mice. BMDM from WT mice (closed bars), IKKε KO mice (open bars), OPTN[D477N] mice (striped bars) and IKKε KO × OPTN[D477N] mice (hatched bars) were stimulated with 10 µg/ml poly(I:C) (**A** and **C**) or 100 ng/ml LPS (**C** and **D**) for the times indicated. (**A** and **B**) The IFNβ mRNA produced was measured relative to 18S ribosomal mRNA and normalized to the level of wild-type IFNβ mRNA (100%) measured after 2 h stimulation with poly(I:C) (**A**) or 1 h stimulation with LPS (**B**). (**C** and **D**) The amount of IFNβ in the cell culture medium was measured by ELISA after stimulation for 8 h with poly(I:C) (**C**) or 4 h with LPS (**D**). (**A**–**D**) The results are presented as arithmetic mean (±SEM for three independent experiments carried out on BMDM from eight different mice).

LPS stimulation decreased the electrophoretic mobility of TANK and OPTN (Figure 2). This is caused by the phosphorylation of these proteins, since it was reversed by treatment with phage λ phosphatase (Supplementary Figure S3). The LPS-induced decrease in the electrophoretic mobility of OPTN is less pronounced in the OPTN[D477N] mutant (Figure 2, panel 6 from top), indicating that it is phosphorylated less extensively than wild-type OPTN. The OPTN[D477N] mutant was also expressed at higher levels than wild-type OPTN. After stimulation with poly(I:C) for 1 or 2 h, there is an apparent decrease in the expression of TANK, which is largely restored after 4 h. The underlying molecular mechanism is unclear, but a possible explanation is that TANK undergoes a covalent modification that prevents its recognition by the TANK antibody.

The LPS- and poly(I:C)-dependent phosphorylation of the MAP kinases, termed c-Jun N-terminal kinases 1 and 2 (JNK1/2) and p38α, was similar in BMDM from TANK KO × OPTN[D477N] and wild-type mice up to 1 h. However, the poly(I:C)-dependent activation of these MAP kinases then decreased much more rapidly between 1 and 4 h in TANK KO × OPTN[D477N] BMDM compared with wild-type BMDM (Figure 2A,B, panels 9 and 10 from top). These observations, which were made in two independent experiments, suggest that these TBK1 heterodimers may have a role in maintaining JNK1/2 and p38α activity during prolonged TLR3 activation. This could be important for sustaining *ifn*β gene transcription in mouse BMDM, since the JNK-dependent activation of the transcription factors c-Jun and ATF2 has been reported to co-ordinate the adenovirus-mediated induction of primary IRF3-responsive transcripts in conjunction with activated IRF3 [43].

### Activation of the TANK–TBK1 and OPTN–TBK1 complexes

**Figure 4.**
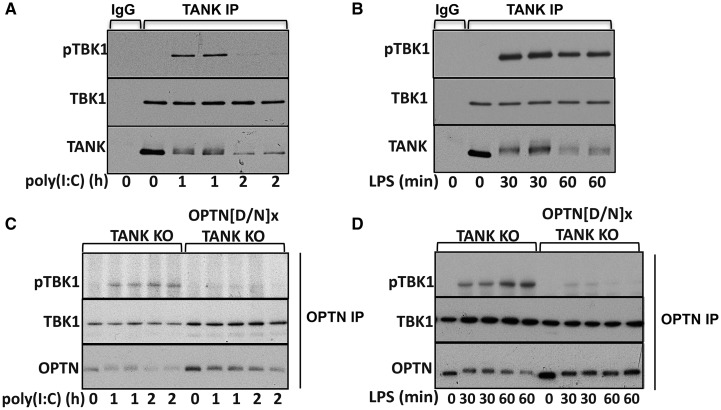
Poly(I:C)- or LPS-dependent activation of the individual TANK–TBK1 and OPTN–TBK1 complexes in BMDM. (**A** and **B**) WT BMDMs were stimulated with 10 µg/ml poly(I:C) (**A**) or 100 ng/ml LPS (**B**) for the times indicated in Figure 1. TANK was immunoprecipitated from the extracts and phosphorylation of the TBK1 in the immunoprecipitates (pTBK1) was analyzed by immunoblotting as described in Materials and Methods. The membranes were also immunoblotted with antibodies that recognize all forms of TBK1 and TANK. (**C** and **D**) As in **A**, **B** except that OPTN was immunoprecipitated from the extracts of TANK KO and TANK KO x OPTN[D477N] mice and the presence of phospho-TBK1, total TBK1 and OPTN in the immunoprecipitates was analyzed by immunoblotting.

To study the activation of the individual TANK–TBK1 and OPTN–TBK1 complexes, we immunoprecipitated TANK (Figure 4A,B) or OPTN (Figure 4C,D) from BMDM extracts and examined phosphorylation of the associated TBK1 catalytic subunit at Ser172. These experiments showed that poly(I:C)- or LPS stimulation induced the activation of both the TANK–TBK1 and OPTN–TBK1 complexes. Importantly, the phosphorylation of TBK1 at Ser172 was reduced in the OPTN[D477N]–TBK1 complex compared with that in the OPTN–TBK1 complex (Figure 4C,D), implying that the interaction of OPTN with ubiquitin chains is required for the robust phosphorylation and activation of the OPTN–TBK1 complex.

### TANK–TBK1 and OPTN–TBK1 are not rate-limiting for Sendai virus-induced IRF3 phosphorylation and IFNβ gene transcription in BMDM

To investigate whether the TANK–TBK1 and OPTN–TBK1 complexes were rate-limiting in the RLR-dependent signalling pathway (see Introduction), we studied Sendai virus-induced IFNβ production in BMDM. These experiments showed that IRF3 phosphorylation at Ser396 and IFNβ secretion were similar in BMDM from TANK KO, OPTN[D477N], TANK KO × OPTN[D477N] and wild-type mice (Supplementary Figure S4). Therefore, in contrast with the TLR3- and TLR4-signalling networks, the TANK–TBK1 and OPTN–TBK1 complexes were not rate-limiting for RLR-dependent IFNβ production under the conditions that were studied.

### The poly(I:C)-dependent phosphorylation of TBK1 at Ser172 in HACAT cells involves a novel ‘upstream’ kinase

The interleukin-1 (IL-1)-dependent phosphorylation of TBK1 at Ser172 in mouse embryonic fibroblasts (MEFs) is catalyzed by IKKβ and by TBK1 itself, and is therefore prevented by incubating IKKα-deficient MEFs with an IKKβ inhibitor plus a dual TBK1/IKKε inhibitor, but not by either inhibitor alone. Since the IL-1-dependent activation of IKKα and IKKβ in MEFs is catalyzed by the protein kinase TAK1 (also called MAP3K7), the IL-1-dependent phosphorylation of TBK1 can also be suppressed by a TBK1 inhibitor in MEFs from knockin mice expressing a kinase-inactive form of TAK1 [30]. In contrast, the tumour necrosis factor-induced activation of TBK1 in IKKα-deficient MEFs is prevented by the inhibition of IKKβ alone [30].

**Figure 5.**
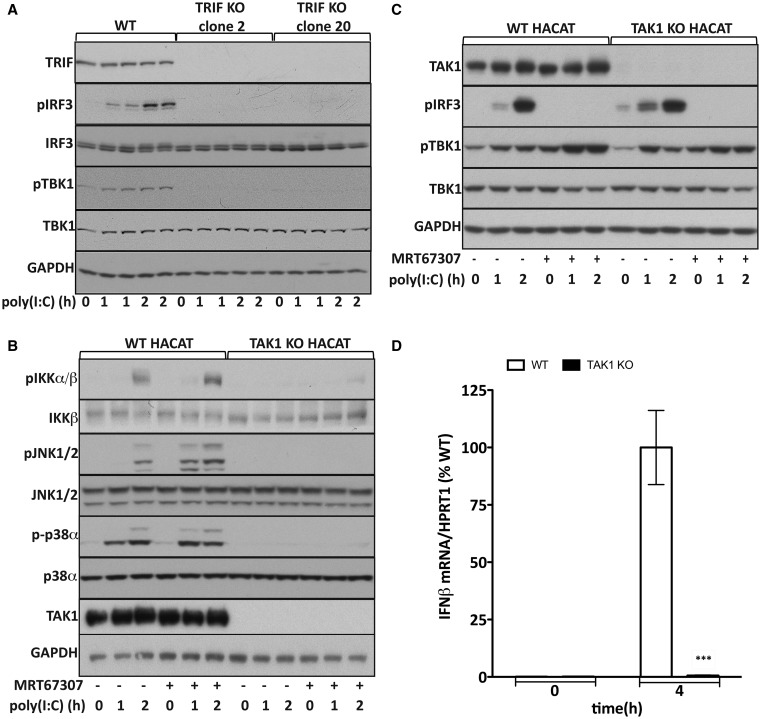
Poly(I:C)-dependent phosphorylation of TBK1 and IFNβ gene transcription in TRIF KO and TAK1 KO HACAT cells. (**A**) Poly(I:C)-dependent phosphorylation of TBK1 and IRF3 is abolished in TRIF KO HACAT cells. Cells were stimulated with 10 µg/ml poly(I:C) for the times indicated as in Figure 1, and the cell extracts (20 µg of protein) were subjected to SDS–PAGE and immunoblotting with antibodies that recognize TBK1 phosphorylated at Ser172 (pTBK1) or IRF3 phosphorylated at Ser396 (pIRF3), and with antibodies that recognize all forms of TBK1, IRF3 and TRIF. Antibodies to GAPDH were used as a loading control. (**B**) TAK1 KO and WT HACAT cells were incubated for 1 h with 2.0 µM MRT67307 and then stimulated with 10 µg/ml poly(I:C) for the times indicated. The cell extracts were processed as in **A** and immunoblotted with antibodies that recognize the phosphorylated (p), activated forms of IKKα/β, JNK1/2 and p38α MAP kinase (p38α) and all forms of TAK1, IKKβ, JNK and p38α. (**C**) The TAK1 KO and WT cells were incubated for 1 h with or without 2 µM MRT67307 prior to stimulation with poly(I:C). Other details are as in **A**,**B**. (**D**) WT HACAT cells (open bars) and TAK1 KO HACAT cells (closed bars) were stimulated with poly(I:C). IFNβ mRNA production was then measured relative to hypoxanthine-guanine phosphoribosyltransferase 1 (HPRT1) mRNA and normalized to the level of IFNβ mRNA measured in WT cells (100%) after stimulation for 4 h with poly(I:C). The results are presented as arithmetic mean (±SEM for two independent experiments each performed in triplicate).

MEFs do not respond robustly to either poly(I:C) or LPS, and TAK1 KO/knockin mice display early embryonic lethality. To investigate which protein kinases activate TBK1 in the TLR3/TLR4–TRIF signalling network, we therefore used human keratinocyte HACAT cells, which respond to poly(I:C) (Figure 5A) [44]. The poly(I:C)-dependent phosphorylation of IRF3 at Ser396 in HACAT cells was mediated by the activation of TLR3, since it was abolished by the KO of TRIF (Figure 5A). We next disrupted the gene encoding the TAK1 catalytic subunit in HACAT cells by clustered regularly interspaced short palindromic repeats (CRISPR)/Cas9 gene-editing technology and found that the poly(I:C)-dependent activation of the canonical IKK complex, JNK1/2 and p38α MAP kinase was abolished (Figure 5B), but the phosphorylation of IRF3 at Ser396 was unaffected (Figure 5C). However, the phosphorylation of TBK1 at Ser172 was only reduced modestly, even when the TAK1 KO cells were additionally incubated with the dual TBK1/IKKε inhibitor MRT67307 prior to stimulation with poly(I:C) to prevent the autophosphorylation of TBK1 at Ser172 (Figure 5C). Taken together, these experiments indicate that the poly(I:C)-dependent phosphorylation of TBK1 at Ser172 involves a novel TBK1-activating kinase distinct from IKKα, IKKβ and TBK1 itself. Incubation of the wild-type HACAT cells with MRT67307 increased the poly(I:C)-dependent phosphorylation of TBK1 at Ser172 (Figure 5C). This observation, which has been made previously in other cells [30], indicates that TBK1 controls a feedback loop that restricts its own activation.

Interestingly, although the poly(I:C)-dependent phosphorylation of IRF3 at Ser396 was unimpaired in TAK1 KO HACAT cells (Figure 5C), IFNβ gene transcription was abolished (Figure 5D), demonstrating that TAK1 controls IFNβ gene transcription by a mechanism that is independent of IRF3 phosphorylation at Ser396. This might be explained by the suppression of JNK1/2 in TAK1 KO HACAT cells (Figure 5B), since JNKs have been reported to control IFNβ gene transcription by phosphorylating IRF3 at Ser173 [45].

## Discussion

Although it is well established that the activation of TBK1 and the phosphorylation of IRF3 are key events in the TLR3/4 signalling pathway leading to IFNβ gene transcription, the molecular events that trigger the activation of TBK1 and the phosphorylation of IRF3 are still incompletely understood. Here, we have shown that the TANK–TBK1 and OPTN–TBK1 complexes are rate-limiting for the TLR3/4-dependent phosphorylation of IRF3 (Figure 2A,B) and *ifnb* gene transcription (Figure 1), whereas the TANK–IKKε heterodimer is not (Figures 2C,D and 3). However, our results do not exclude roles for other TBK1 heterodimers in this pathway, such as the NAP1–TBK1 and SINTBAD–TBK1 heterodimers which might, for example, mediate the TBK1-catalyzed activation of other proteins that control this process, such as DDX3X [14,15]. Moreover, the TANK–TBK1 and OPTN–TBK1 heterodimers were not rate-limiting for Sendai virus-induced IRF3 phosphorylation and IFNβ secretion, indicating the involvement of additional/other TBK1 heterodimers in the RIG-I/MDA-5 pathway (Supplementary Figure S4). Our experiments have also revealed that the poly(I:C)/TRIF-dependent phosphorylation of TBK1 at Ser172 in human HACAT cells involves a protein kinase(s) distinct from or additional to the canonical IKK complex and TBK1 itself (Figure 5), which are the protein kinases known to phosphorylate TBK1 at Ser172 in the IL-1- and TNF-signalling pathways [30].

We observed that the poly(I:C)- or LPS-induced phosphorylation of Ser172 in the OPTN–TBK1 complex is prevented in the OPTN[D477N]–TBK1 heterodimer (Figure 4), implying that the interaction of ubiquitin chains with OPTN is required to activate the TBK1 catalytic subunit in this complex. Consistent with this finding, the poly(I:C) or LPS-stimulated phosphorylation of OPTN was reduced in macrophages expressing the ubiquitin-binding-defective OPTN[D477N] mutant (Figure 2); TBK1 is known to phosphorylate OPTN at Ser177 [35,46]. The activation of the OPTN–TBK1 complex in the TLR3/4 signalling pathway therefore appears to resemble the IL-1-dependent activation of the NEMO–IKKβ complex where the formation of Met1-linked ubiquitin chains (catalyzed by the linear ubiquitin assembly complex) and their binding to NEMO are required before TAK1 can phosphorylate the activation loop of IKKβ at Ser177 [47]. In the IL-1 signalling pathway, the Met1-linked ubiquitin oligomers are attached covalently to preformed Lys63-linked ubiquitin oligomers, providing a mechanism for the co-recruitment of the canonical IKK complex and its activator TAK1 [48]. Hybrid ubiquitin chains containing both Met1- and Lys63-ubiquitin linkages are also formed when the TLR3–TRIF signalling pathway is activated [49]. It is therefore tempting to speculate that these hybrid ubiquitin chains recruit the OPTN–TBK1 complex to its as yet unknown activating kinase.

## Abbreviations

BMDM: bone marrow-derived macrophages
CRISPR: clustered regularly interspaced short palindromic repeats
ds: double-stranded
IFN: interferon
IL-1: interleukin-1
IKK: IκB kinase
IRF3: interferon regulatory factor 3
JNK1/2: c-Jun N-terminal kinases 1 and 2
KO: knockout
LPS: lipopolysaccharide
MAP: mitogen-activated protein
MAVS: mitochondrial antiviral signalling protein
M-CSF: macrophage colony-stimulating factor
MDA-5: melanoma differentiation-associated protein 5
MEF: mouse embryonic fibroblast
MyD88: myeloid differentiation primary response gene 88
NAP1: NF-κB-activating kinase-associated protein 1
NEMO: NF-κB essential modulator
OPTN: optineurin
PCR: polymerase chain reaction
RLRs: RIG-I-like receptors
SINTBAD: Similar to NAP1 TBK1 Binding Adaptor
TAK1: TGFβ-activated kinase-1
TBK1 TANK: binding kinase 1
TRAF-: tumour necrosis factor (TNF) receptor associated factor
TLR: Toll-like receptor
TANK: TRAF family member-associated NF-κB activator
TRIF: TIR-domain-containing adapter-inducing IFNβ.
